# Situation analysis on the regulation of nanomedicines in Southern Africa

**DOI:** 10.3389/fmed.2023.1098830

**Published:** 2023-04-20

**Authors:** Linda G. Mudyiwenyama, Star Khoza, Admire Dube

**Affiliations:** School of Pharmacy, University of the Western Cape, Bellville, South Africa

**Keywords:** regulation of nanomedicines, African Medicines Agency, regulatory workforce capacity building, nanomedicine, ZaZiBoNA, medical products regulation

## Abstract

**Background:**

Medical products incorporating nanoparticle drug delivery systems (nanomedicines) are therapeutic or imaging agents, which comprise a delivery system within the nanometer size range (1 – 1000 nm). As medical products, nanomedicines meet definitions of medicines according to various national legislations for regulation of medicines. However, for the regulation of nanomedicines, additional assessments including toxicological issues have to be considered. These complexities require extra regulatory effort. In the resource-limited context of low- and middle-income countries, many National Medicines Regulatory Authorities (NMRAs) lack resources and capacities to effectively assure the quality of medicinal products in their countries. With emerging trends in innovative technologies, including nanotechnology, this burden is worsened. The need to overcome regulatory challenges drove the formation of a work sharing initiative in the Southern African Development Community (SADC), ZaZiBoNA in 2013. Regulatory agencies participating in this initiative cooperate in the assessment of applications for registration of medicines.

**Methods:**

A cross-sectional exploratory study design with qualitative techniques was used to investigate the status of the regulation of nanomedicines in Southern African countries in particular those participating in the ZaZiBoNA initiative.

**Results:**

The study found that in general, NMRAs are aware of the existence of nanomedicines and they apply legislation applicable to other medical products. The NMRAs however neither have specific definition for nanomedicines and technical guidance documents, nor technical committees specific for consideration of nanomedicines. Collaboration with external experts or organisations in the regulation of nanomedicines was also found to be lacking.

**Discussion:**

Capacity building and collaboration in the area of regulation of nanomedicines is strongly encouraged.

## Introduction

Medical products incorporating nanoparticle drug delivery systems (also usually referred to as nanomedicines) are defined as therapeutic or imaging agents which comprise a delivery system within the nanometer size range (1–1,000 nm) in order to control the drug delivery, uptake and biodistribution, enhance efficacy, or reduce toxicity of the drug (1–3). Currently, several nanomedicines are approved for use worldwide, including products for the treatment of cancers, autoimmune diseases, fungal infections, hepatitis, among other conditions (4). Recently, during the COVID-19 pandemic, innovations in nanotechnology for intracellular delivery and advances in nanomedicine production have recently been used in the production of mRNA-based vaccines for emergency use in vaccination against COVID-19 (5, 6). Additional applications of nanomedicines include use in vaccinations, magnetic resonance imaging (MRI) contrast agents, fluorescent biological labels, pathogen detection, protein identification, DNA structure probing, tissue engineering, drug- and gene-delivery agents, and the separation of biological molecules and cells (7–10). Several more are undergoing clinical trials, with most being investigated for therapy in cancer and infectious diseases (3). Twenty-two ongoing clinical trials involving medicines and diagnostics using nanoparticles with locations in Africa are registered on ClinicalTrials.gov (11). Twenty-five clinical studies which had locations in Africa are also registered as complete on the same site. With regard, studies for Covid-19 vaccines utilizing nanoparticles, four studies registered on ClinicalTrials.gov are located in Africa (11). Clinical investigations to expand the use of approved nanomedicines are also on going, for example investigation of use of VYXEOS®, a combination chemotherapy nanoparticle in additional patient populations and leukemia (12).

The first generation of nanomedicines, mainly liposomes were first approved for marketing in 1990. The United States Food and Drug Administration (USFDA) approved the first pegylated adenosine deaminase enzyme in 1990 (7). Most of the currently approved nanomedicines consist of relatively simple nanoparticles and build on the success of well described nanoparticle systems and prior approved drugs, e.g., PEGlyated liposomal doxorubicin (13). There has been both a broadening in nanoparticle types and an increase in the complexity of nanoparticles within these categories over time (1). This presents challenges for medical product regulators.

As medical products, nanomedicines would meet the definitions of medicines according to various national legislations for medical products regulation. However, for the regulation of nanomedicines critical quality attributes (CQAs) and additional toxicological assessments have to be considered, in addition to the general requirements for medicines. This is due to the wide range of structures of the nanomedicines, their physicochemical and biological properties, and the variety of therapeutic applications that makes the generalization of CQAs a challenge (14). These additional considerations need to be translated into standardized and regulatory accepted test methods, testing strategies, guidelines and policies (15). The USFDA and EMA have developed scientific guidelines on nanomedicines to assist manufacturers to prepare marketing authorization applications for medicines. Guidance documents developed by the EMA include specific guidelines for intravenous iron-based nano-colloidal products, intravenous liposomal products and general considerations for parenteral administration of nanomedicine products (16). Similarly, the USFDA has issued guidance for industry to offer advice, including advice to determine the regulatory status of nanotechnology products and evaluating their safety (17).

In the resource-limited context of low- and middle-income countries (LMICs), many National Medicines Regulatory Authorities (NMRAs) still lack the resources and capacities to effectively assure the quality of medicinal products manufactured, imported or circulating in their territory (18, 19). The African Medicines Regulatory Harmonization (AMRH) initiative is intended to improve the regulatory system for product registration in Africa with a focus on collaborative regulation and forms the basis for the establishment of the African Medicines Agency (AMA). Regional work sharing is therefore encouraged and one such initiative is the ZaZiBoNa work sharing initiative in South Africa (20, 21). Regulatory agencies participating in this initiative therefore cooperate in the assessment of applications for registration of medicines (22, 23).

This study therefore sought to investigate the status of the regulation of nanomedicines in Southern African countries in particular those participating in the ZaZiBoNA work sharing initiative, with a view to identify challenges specific to nanomedicines being encountered as well as documenting priority areas for capacity building and harmonization.

## Methods

### Study design and population

A study sample consisting of regulatory authorities active in the ZAZIBONA joint assessments was used in the questionnaire based, cross-sectional study.

All nine countries participating actively in ZaZiBoNA joint assessments were included in the study, i.e., Botswana, Democratic Republic of Congo, Malawi, Mozambique, Namibia, South Africa, Tanzania, Zambia and Zimbabwe.

### Development and pre-testing of questionnaire

A structured questionnaire was used to collect data, with questions to gather information on awareness of nanomedicines, existence of legal mandate and regulatory framework to regulate nanomedicines. In addition, questions to ascertain regulatory experience with nanomedicines, and areas that were perceived as important for process improvement in the regulation of nanomedicines were included in the questionnaire. The questions included whether the NMRAs had specific definitions for nanomedicines, legal provisions that cover regulation of nanomedicines, guidance documents for submission and assessment of nanomedicines applications, existence of specific technical committee for consideration of advanced medicines including nanomedicines, in-house assessment templates for nanomedicines and if any regional harmonization activities the NMRAs participated involved nanomedicines applications.

The tool was piloted with one country participating in ZAZIBONA as a non-active member. The tool was assessed for volatility, ease of use and comprehensiveness. The pilot country was chosen as it is regularly involved in the ZAZIBONA assessment activities.

Although the country does not participate in ZAZIBONA with an active status, it has been involved with the initiative since November 2016, participating in all relevant meetings.

### Distribution of questionnaire

The questionnaire was sent to the heads of national regulatory authorities of the ZAZIBONA active countries electronically in 2020 and 2021. Reminder emails were sent every 2 weeks, if no response was received. Further email follow-ups were made for 4 months. Thereafter, if no response was received, it was assumed that the country was unwilling to participate in the study.

### Data analysis

Thematic and descriptive analysis were used to describe and summarize data as well as interpret patterns in the responses. Responses to the questions on the questionnaire were reviewed, identifying aspects of data that were interesting and informative in developing themes. Codes were developed from this data by grouping elements of data according to similarities and patterns. The coded data was then reviewed and analyzed to combine codes with shared meanings to form thematic categories for interpretation.

### Ethical consideration

Ethical approval for the study was granted by the Humanities and Social Science Research Ethics Committee of the University of the Western Cape, ethics approval number HS20/3/8. Written consent was also received from participants in the questionnaire.

## Results and discussion

Out of the nine NMRAs that were requested to participate, seven NMRAs responded. Respondents were Botswana Medicines Regulatory Authority (BoMRA), Medicines Control Authority of Zimbabwe (MCAZ), Namibia Medicines Regulatory Council (NMRC), the Pharmacy and Medicines Regulatory Authority (PMRA) of Malawi, South African Health Products Regulatory Authority (SAHPRA), Tanzania Medicines and Medical Devices Authority (TMDA) and Zambia Medicines Regulatory Authority (ZAMRA).

**Figure 1:**
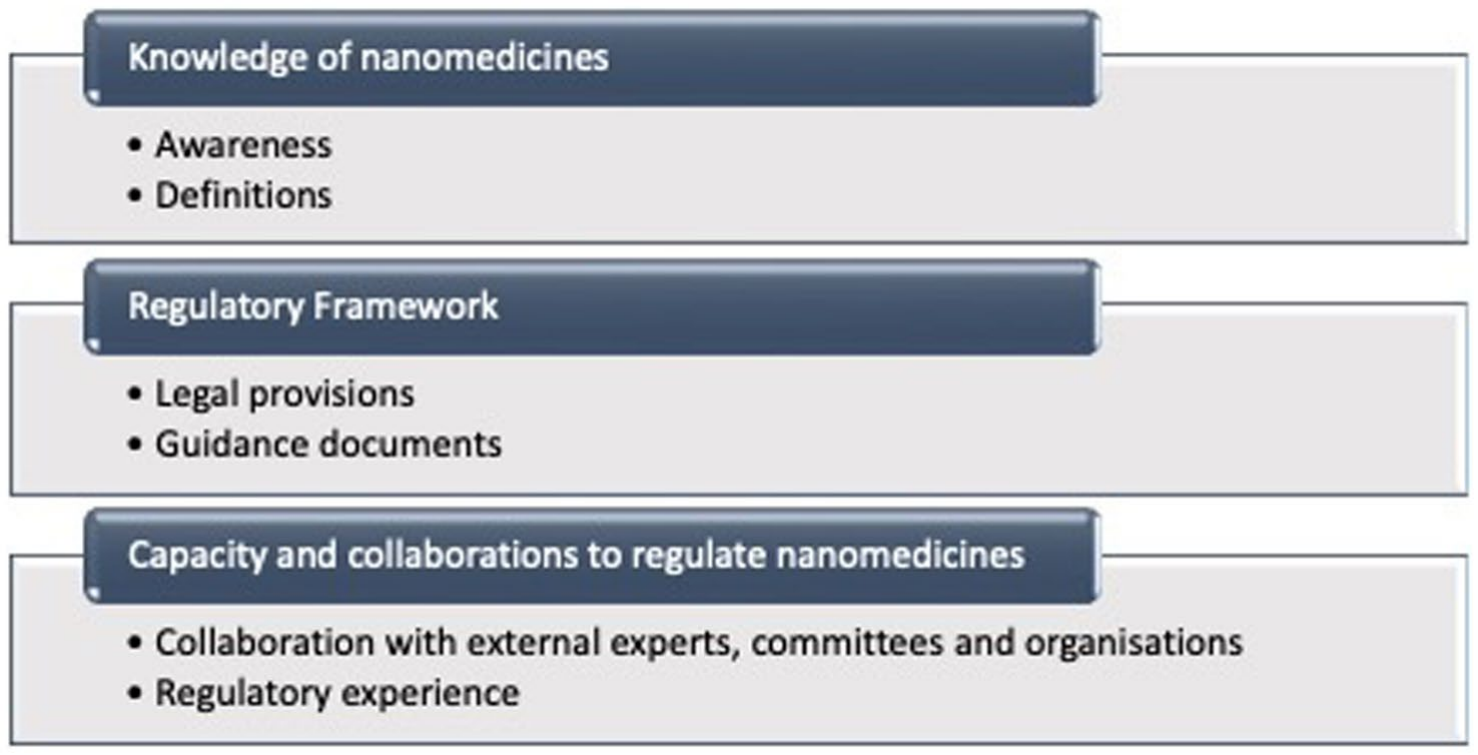
Thematic map.

The responses were collated into three main themes each with two sub-themes. The main themes concluded from this study are Regulatory Framework, knowledge of nanomedicines, capacity and collaborations to regulate nanomedicines. These are highlighted in Figure 1 below.

### Awareness of nanomedicines

Out of seven respondents, one respondent indicated that they were not aware of what nanomedicines were. There was therefore a high level of awareness of nanomedicines among the respondents. Awareness of nanomedicines by medicines regulators is considered worth evaluating as regulators have a privileged knowledge position. As such, they are expected to be aware of trends and emerging technologies related to medicines. Their opinion and awareness are also likely to influence public perception and acceptability of nanomedicines by the general public. Furthermore, awareness, knowledge and understanding of nanomedicines by regulators are important prerequisites for the contribution to the development, implementation and maintenance of effective regulatory systems for these products.

### Legal provisions

Four of the seven respondents from the NMRAs had legal provisions that cover regulation of nanomedicines. The provisions were reported to be the same as those for all medicines regulated by the authorities as they are mandated to regulate all medicines irrespective of the technology applied. Legal provisions give mandate to national regulatory authorities to oversee the regulation of nanomedicines as well as enforcement powers over the requirements. Rather than developing separate legislation specific for nanomedicines, the situation within the NMRAs is expected to streamline and simplify the regulatory process for nanomedicines, as they will be incorporated into the already existing structures of regulation. The USFDA also adopted a similar approach. They indicated that nanomedicine is not different to any other new technology that is incorporated into FDA products (24–26). As such, there was no need for regulations written specifically for nano-engineered materials in the products regulated by FDA (17). Likewise, EMA through its AdHoc Informal Group on nanomedicines highlighted that new set of guidelines for nanomedicines was not necessary; rather integration in the existing regulatory framework had to be considered (27).

Of the three respondents that did not have legal provisions covering regulation of nanomedicines, two had not received applications for approval of any of the USFDA and EMA approved nanomedicines listed in the questionnaire. Lack of receipt of such applications could have been because the authorities had no mandate to regulate nanomedicines. The opposite could also be considered possible; the authorities may have not seen the need to develop legislation for the regulation of nanomedicines as they had not received applications for registration of such products. For the other respondent who indicated that their regulatory agency did not have provisions that covered nanomedicines, that their regulatory agency had received the highest number of applications for nanomedicines in comparison with the other NMRAs in the survey. Also, the same respondent indicated that they were a member of the of the International Pharmaceutical Regulators Programme (IPRP) and they were in the process of developing guidance documents for applicants as well as in-house guidance documents to assist with assessment of nanomedicines. The negative response regarding legal provisions for regulation of nanomedicines could therefore had been an oversight on the respondent’s part or because the NMRA was in the process of revising their legislation to include regulation of nanomedicines.

### Definition of nanomedicines

None of the respondents from the NMRAs indicated existence of a specific definition for nanomedicine within their NMRAs. Their definition for nanomedicines however, was within the general definition for medicines. These observations are similar to the approach taken by the USFDA and EMA who do not have a formal regulatory definition for nanomaterials, nanoscale, nanotechnology or nanomedicine (17, 28). The USFDA took a broad, inclusive approach by determining whether the products they regulate contain nanomaterials or whether they involve nanotechnology (17, 29). Globally, there is also no consistent and uniform definition of nanomedicines. For example, the US National Nanotech Initiative in their definition for nanomedicines clearly refer to the nanoscale (1–100 nm). On the other hand, the European Science Foundation and the European Technology Platform on Nanomedicine do not refer to it (30). In the context of SADC medicines regulation, NMRAs in the ZaZiBoNa active countries should consider coming up with a working definition for nanomedicines. This could facilitate effective regulation of nanomedicines. With a clear definition the risk of miscommunication with various stakeholders is minimized. Working definitions adopted by the other jurisdictions could be considered.

### Guidance documents

None of the NMRAs had specific guidance documents which cover submission of quality, non-clinical/safety and clinical information for applications for nanomedicines. However, one NMRA indicated that it is a member of the IPRP and therefore, applies that the principles, guidance documents and templates as laid out by the IPRP for nanomedicines. Furthermore, the same regulatory agency stated that they use guidance documents from the EMA. Lack of specific guidance documents which cover submission of quality, non-clinical/safety and clinical information for applications for nanomedicines in all the responding NMRAs is likely to impair applicants’ and assessors’ capacity to deal with the inherent uncertainty surrounding requirements for applications of nanomedicines. Harmonized guidance documents to facilitate approval of these nanomedicines and ease the application process could be developed to assist applicants in submitting applications for registration in the SADC region. Establishment of the AMA may also possibly address such issues by implementing agreed procedures and processes and coordinating regulatory practices across the region. Such coordinated regulatory and pooled procurement efforts could motivate manufacturers and marketing authorization holders to supply the innovative nanomedicines to SADC countries.

Jurisdictions in other regions of the world have developed guidelines specific for nanomedicines. The EMA, for example, has developed scientific guidelines on nanomedicines to assist manufacturers to prepare marketing authorization applications for human medicines (16). Similarly, the USFDA has issued guidance for industry to offer advice, including advice to determine the regulatory status of nanotechnology products and evaluating their safety (17, 29).

Two of the seven NMRAs in this study indicated that they were in the process of developing specific guidance documents for submission of information for applications for nanomedicines. One of these NMRAs had received two applications for market approval for nanomedicines, while the other had received 11 applications for market approval. Development of guidance documents to outline submission requirements for nanomedicines applications is thus plausible, as these will assist applicants with the requirements for approval of nanomedicines.

None of the NMRAs had in-house guidance documents for the assessment of quality, non-clinical/safety and clinical aspects of nanomedicines and two indicated that they were in the process of developing such guidance documents. Correspondingly, none of the NMRAs had assessment templates specific for nanomedicines and two were in the process of developing such guidance documents. NMRAs should develop internal guidance documents that complement the relevant existing guidelines such that pertinent issues related to nanomedicines are not disregarded. This opinion is supported by the *Agence française de securite sanitaire des produits de sante*’s position in which they considered that toxicological evaluation of nanomedicines should not be appreciably different from conventional evaluation, but with certain specific adaptations when necessary, without modifying the basic principle (31).

### Collaboration with external experts, committees, and organizations

The common practice among national medicines regulatory authorities, is the use of technical committees to provide expert advice on subject matters. The technical committees make decisions to authorize or not authorize medicines based on available data concerning the safety, effectiveness and quality of the medicines. It is therefore important for the committees to have the necessary expertise on the product type under consideration. One NMRA indicated that it has a specific technical committee for consideration of advanced drug delivery systems including nanomedicines or committee members with expertise in nanomedicines. This is the same agency that has been noted to be advanced in terms of nanomedicines assessment as it has received the highest number of nanomedicines applications for market approval, and has been involved with the IPRP.

To provide expertise that may be lacking in the regulation of nanomedicines within their organizations and consequently effectively regulate nanomedicines, NMRAs could ensure that external experts involved in their marketing authorization decision making processes include personnel who have extensive expertise in issues related to safety, efficacy and quality of nanomedicines. This could be through creating a separate committee for discussion of advanced drug delivery systems including nanomedicines. However, since the number of product applications may not justify such an approach; personnel with expertise in such matters could be co-opted into the already existing committees to provide advice as and when required. On the other hand, the NMRAs may also use a system whereby external experts conduct the review of all or part(s) of the applications for nanomedicines.

Regulatory cooperation and work-sharing is important, especially in the complex area of nanomedicines. In response to a question on whether nanomedicines are considered under the regional harmonization activities that the responding NMRAs are involved in, it was established that currently no assessments of nanomedicines applications are considered under the ZaZiBoNa regional harmonization activities. In addition to domestic collaborations, the NMRAs could also consider interacting with regulatory bodies in other countries in order to remain current with product registration going on internationally, since these products may be submitted to their NMRAs for review and approval in future.

### Regulatory experience with nanomedicines

**Figure 2:**
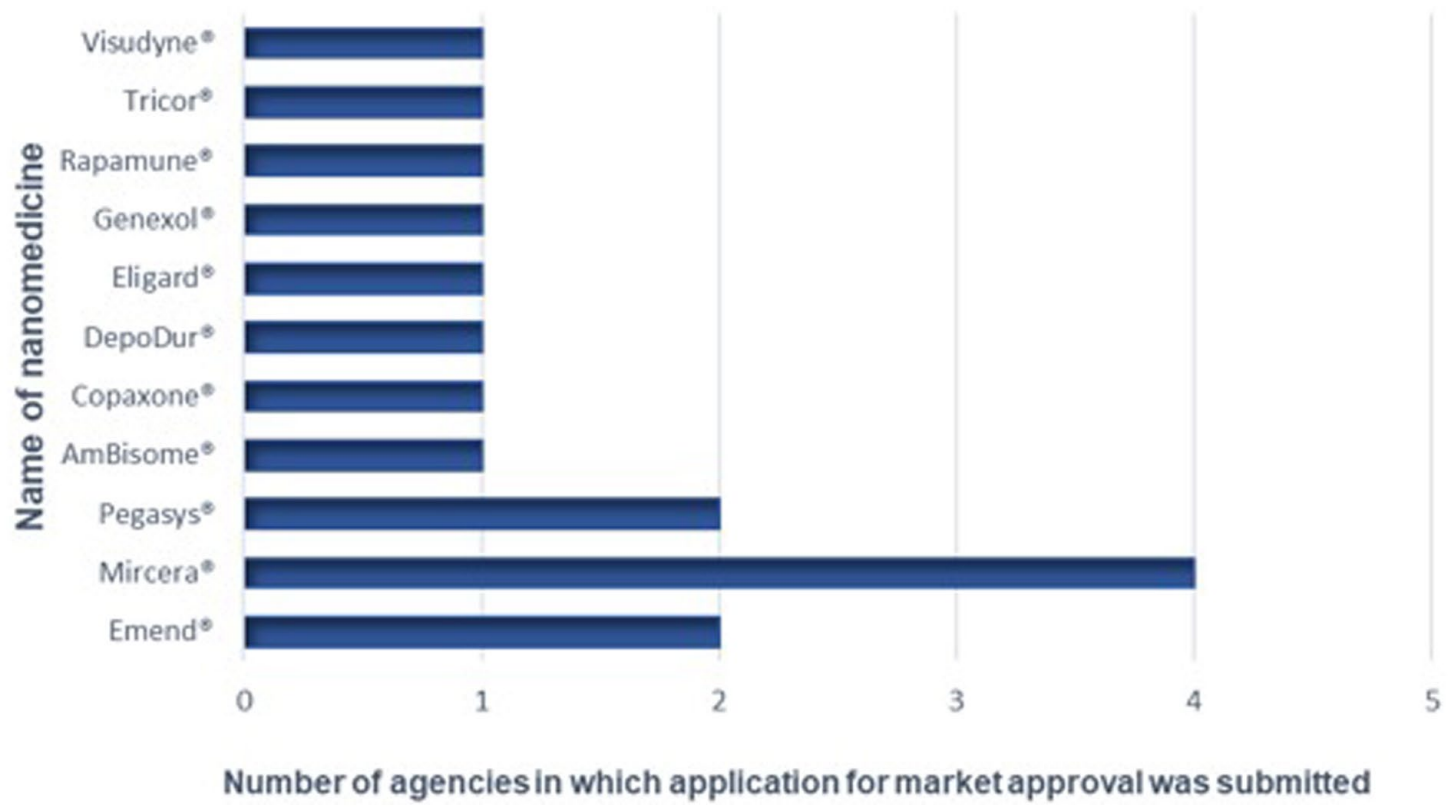
Nanomedicines for which applications for marketing approval have been submitted to the NRAs.

The respondents were presented with a list of USFDA and EMA approved nanomedicines and asked to identify products for which applications for registration or approval had been submitted to their NMRAs in the last 10 years. Four NMRAs responding had received at least one application for approval of nanomedicines in the last 10 years whilst three had not received any applications for registration for nanomedicines in the last 10 years. One agency had received 11 applications for market approval of nanomedicines in the last 10 years, another one had received three applications, followed by an agency that had received two applications for such medicines and one agency had received one application for market approval of a nanomedicine (Figure 2).

The most commonly received nanomedicine was Mircera®, which was received by four of the NMRAs. Mircera® is a solution for injection that contains the active substance Epoetin beta (as methoxy polyethylene glycol-epoetin beta conjugate) (32) and ^is^ indicated for the treatment of symptomatic anemia associated with chronic kidney disease (CKD) in adult patients. Approximately 75% of CKD patients are reported to be anemic in low- and middle-income countries (LMICs) (33). This may explain why Mircera® is the commonly received product for registration in the NRAs that participated in this study. When compared to the number of approved nanomedicines, the numbers submitted to these sub-Saharan African countries appear relatively low. The USFDA is reported to have approved commercialization of at least 100 nanomedicine applications and products (34). This observation could be attributed to the small financial market for nanomedicines in Africa. This situation has been proven by the accessibility of nano-based COVID-19 vaccines authorized for emergency use to prevent COVID-19. Three out of four COVID-19 vaccines procured by the well-resourced countries by the end of 2020 were nanoparticle-based vaccines while one in 10 vaccines were nanoparticle based vaccines in the procured stocks of the middle-income countries. In addition, only one in 285 vaccine stock secured by the COVAX initiative for immunization of people in the world’s poorest countries throughout the first half of 2021 were nanoparticle based vaccines (35).

### Areas for improvement

Respondents from the NMRAs all indicated that there was need for training medicines assessors on assessment of nanomedicines. To improve the internal competencies in the area of nanomedicines as well as address the rapidly evolving regulatory science challenges associated with nanomedicines, NMRAs should invest in nanomedicine specific trainings that bring about both practical and theoretical knowledge of nanomedicines. Additionally, the assessors should have the opportunity to attend relevant conferences, courses and international meetings so that they are aware of international standards associated with regulation of nanomedicines. Another approach that can be taken in building the core competencies necessary for the assessment of nanomedicines is to hire assessors that are scientifically and academically trained in the area of nanotechnology.

In response to a question regarding need for incorporation of assessments in regional harmonization activities the respondents are involved in, all the respondents agreed that there was need, with four out of seven strongly agreeing that there was such need. Regulatory cooperation and work-sharing is important, especially in the complex area of nanomedicines. To leverage resources and other NMRAs’ work, as well as to prevent redundant work in the regulation of nanomedicines, the NMRAs can consider inclusion of nanomedicines into the already existing framework of the ZaZiBoNa joint assessments. This could streamline the assessment of nanomedicines as well as facilitate open dialog among the NMRAs on how they can collaborate to advance scientific understanding of nanomedicines.

## Conclusion

This study assessed the regulatory experience with nanomedicines within the ZaZiBoNa active countries, analyzed their legislation, guidelines and policies on nanomedicines, reviewed assessment practices with respect to applications for nanomedicines, as well as identified challenges and possible opportunities for harmonization with regards to nanomedicines. This study found that in general NMRAs are aware of the existence of nanomedicines and they apply legislation applicable to other medical products. The NMRAs also do not have specific definition for nanomedicines. Most NMRAs do not have a specific technical committee for consideration of advanced drug delivery systems including nanomedicines. Collaboration with external experts or organizations in the regulation of nanomedicines is lacking in the participating NMRAs. Respondents indicated of the need for training and capacity building in the area of assessment of nanomedicines as well as incorporation of nanomedicines assessments in regional harmonization activities. It is proposed that nanomedicines specific guidance documents and templates will be implemented to complement the relevant existing guidelines and pertinent aspects will be assessed as nanomedicines applications for market approval are submitted to the NMRAs. Capacity building and collaboration including work sharing among assessors at NRAs is also strongly encouraged.

## Data availability statement

The raw data supporting the conclusions of this article will be made available by the authors, without undue reservation.

## Ethics statement

The studies involving human participants were reviewed and approved by Humanities and Social Science Research Ethics Committee of the University of the Western Cape, ethics approval number HS20/3/8. The patients/participants provided their written informed consent to participate in this study. The participants also provided their consent by responding to the questionnaire.

## Author contributions

All authors listed have made a substantial, direct, and intellectual contribution to the work and approved it for publication.

## Conflict of interest

The authors declare that the research was conducted in the absence of any commercial or financial relationships that could be construed as a potential conflict of interest.

## Publisher’s note

All claims expressed in this article are solely those of the authors and do not necessarily represent those of their affiliated organizations, or those of the publisher, the editors and the reviewers. Any product that may be evaluated in this article, or claim that may be made by its manufacturer, is not guaranteed or endorsed by the publisher.
