# Supplementary material for: Situation analysis on the regulation of nanomedicines in Southern Africa
Source: Front Med (Lausanne). 2023 Apr 20;10:1098830. doi: 10.3389/fmed.2023.1098830 (PMC10157474; doi:10.3389/fmed.2023.1098830)
Supplement: Supplementary file 1 [file Data_Sheet_1.PDF]

QUESTIONNAIRE

1. Name of National Medicines Regulatory Authority (or equivalent)

____

2. Name and position of respondent

____

PART A: REGULATORY FRAMEWORK

1. Are you aware of the existence nanomedicines? A nanomedicine is defined as a product that contains or is manufactured using materials in the nanoscale range, i.e. 1 nanometer to 100 nanometers,and includes liposomes and other engineered particles in this size range.

☐ Yes ☐ No

Nanomedicine
1. Abelcet®- Amphotericin B complex 1:1 with DMPC and DMPG (7:3), >250 nm, ribbon like structures of a bilayered membrane
2. Adagen®- PEGylated adenosine eaminase. One enzyme molecule is odified with up to 17 strands of PEG, MW 5,000, 114 oxymethylene groups per strand
3. AmBisome® - Amphotericin B encapsulated in liposomes (60–70 nm)omposed of hydrogenated soy phosphatidylcholine, cholesterol, and distearoyl phosphatidylglycerol (2/0.8/1 molar)
4. Amphotec® - Amphotericin B complex with cholesteryl sulfate (1:1). Colloidal dispersion of disc-like particles, 122 nm ×4 nm
5. Cimzia® - PEGylated antibody (Fab' fragment of a humanized anti-TNF-alpha antibody)
6. Copaxone® - Polypeptide (average MW 6.4 kDa) composed of four amino acids (glatiramer)
7. DaunoXome® - Daunorubicin citrate encapsulated in liposomes (45 nm) composed of distearoyl phosphatidylcholine and cholesterol (2/1 molar)
8. DepoCyt® - Cytarabine encapsulated in multivesicular liposomes (20 μm; classified as nanopharmaceutical based on its individual drug containing “chambers”) made from dioleoyl lecithin, dipalmitoyl phosphatidylglycerol, cholesterol, and triolein
9. DepoDur® - Morphine sulfate encapsulated in multivesicular liposomes (17–23 μm; per se not a nanopharmaceutical – classified as such based only on its individual drug containing “nano-sized chambers”) made from dioleoyl lecithin cholesterol, dipalmitoyl phosphatidylglycerol, tricaprylin, and triolein

10. Doxil® - Doxorubicin hydrochloride encapsulated in Stealth® liposomes (100 nm) composed of N-(carbonyl- methoxypolyethylene glycol 2000)-1,2-distearoyl-sn-glycero3-phosphoethanolamine sodium, fully hydrogenated soy phosphatidylcholine, and cholesterol
11. Eligard® - Leuprolide acetate (synthetic GnRH or LH-RH analog) incorporated in nanoparticles composed of PLGH copolymer (DL-lactide/glycolide; 1/1, molar)
12. Emend® - Aprepitant as nanocrystal
13. Genexol® - Paclitaxel in 20–50 nm micelles composed of block copolymer poly(ethylene glycol)- poly(D,L-lactide)
14. Inflexal® V - Influenza virus antigens (hemagglutinin, neuraminidase) on surface of 150 nm Liposomes
15. Macugen® - PEGylated anti-VEGF aptamer
16. Marqibo® - Vincristine sulfate encapsulated in sphingomyelin/cholesterol (60/40, molar) 100 nm liposomes
17. Megace ES® - Megestrol acetate as nanocrystal
18. Mepact™ - Mifamurtide (synthetic muramyl tripeptide-phosphatidylethanolamine) incorporated into large multilamellar liposomes composed of 1-palmitoyl-2-oleoyl-sn-glycerol-3-phosphocholine and 1,2-dioleoyl-sn- glycero-3-phospho-L-serine
19. Mircera® -PEGylated epoetin beta (erythropoietin receptor activator)
20. Myocet®- Doxorubicin encapsulated 180 nm oligolamellar liposomes composed of egg phosphatidylcholine/cholesterol (1/1, molar)
21. Neulasta® - PEGylated filgrastim (granulocyte colony-stimulating factor)
22. Oncaspar® - PEGylated L-asparaginase
23. Opaxio® - Paclitaxel covalently linked to solid nanoparticles composed of polyglutamate
24. Pegasys® - PEGylated interferon alfa-2b
25. PegIntron® - PEGylated interferon alfa-2b
26. Rapamune® - Rapamycin (sirolimus) as nanocrystals formulated in tablets
27. Renagel®- Cross-linked poly allylamine hydrochloride, MW variable
28. Somavert® - PEGylated human growth hormone receptor antagonist
29. Tricor® - Fenofibrate as nanocrystals
30. Triglide®- Fenofibrate as insoluble drug-delivery microparticles
31. Visudyne® - Verteporfin in liposomes made of dimyristoyl-phosphatidylcholine and egg phosphatidylglycerol (negatively charged); lyophilized cake for reconstitution
32. Zinostatin stimalamer® - Conjugate protein or copolymer of styrene-maleic acid and an antitumor protein NCS

33. From the list of USFDA and EMA approved nanomedicines (listed above), please identify products for which applications for registration or approval have been submitted to your regulatory agency in the last 10 years? Please indicate 'None' if no applications have been received.

____

____

____

3. Does your regulatory agency have a definition for nanomedicines?

☐ Yes ☐ No

If you responded YES to the above question, please provide the definition

____

____

____

4. Does your regulatory agency have legal provisions that cover regulation of nanomedicines?

☐ Yes ☐ No

If you responded YES to the above question, please provide details of the legislation.

____

____

____

5. Does your regulatory agency have specific guidelines for submission of quality, non-clinical/safety and clinical information for applications for nanomedicines?

☐ Yes ☐ No

If your regulatory agency has specific guidance documents for submission of quality, non-clinical/safety and clinical information for applications, including nanomedicines, please provide links to the guidance documents, if publicly available.

____

If your regulatory agency does not have specific guidance documents for submission of quality, non-clinical/safety and clinical information for applications, including nanomedicines; is your regulatory agency in the process of developing such guidance documents?

☐ Yes ☐ No

6. Does your regulatory agency have in-house guidelines for the evaluation of the quality, non-clinical/safety and clinical aspects of nanomedicines?

   ☐ Yes ☐ No

   If you regulatory agency does not have in-house guidance documents for the assessment of nanomedicines, is your regulatory agency in the process of developing such guidance documents?

   ☐ Yes ☐ No

7. Does your regulatory agency have a specific technical committee for consideration of advanced drug delivery systems including nanomedicines or committee members with expertise in nanomedicines?

   ☐ Yes ☐ No

8. Please specify other external experts or organisations that assist your regulatory agency with regulation of nanomedicines, if any.

____

9. Have you or anyone in your organization received training on assessment of advanced drug delivery systems including nanomedicines?

☐ Yes ☐ No

10. Does your regulatory agency have assessment templates specific for nanomedicines?

☐ Yes ☐ No

11. Does your nanomedicines specific template(s) or any of your assessment templates cover assessment of the following with respect to nanomedicines? (You may tick more than one response)

☐ Physicochemical characterisation
☐ Characterisation methods
☐ Biological characterisation
☐ Ecotoxicology
☐ Toxicity testing
☐ Stability
☐ Endotoxin assessment

12. Are any assessments of nanomedicines applications considered under regional harmonisation activities you are involved in?

☐ Yes ☐ No

If you responded YES to the above question, please specify the regional harmonisation activities

____

____

____

PART B: AREAS FOR IMPROVEMENT

13. In your own opinion, is there need for training assessors on assessment of nanomedicines?

☐ Strongly agree
☐ Agree

- ☐ Disagree
- ☐ Strongly disagree
- ☐ Unable to say

14. In your own opinion, is there need to incorporate assessment of nanomedicines into the regional harmonisation activities?
    - ☐ Strongly agree
    - ☐ Agree
    - ☐ Disagree
    - ☐ Strongly disagree
    - ☐ Unable to say

15. Is there anything additional that you would like to mention with regards to this topic or questions above.

    ____

    ____

    ____

    ____
